# The Role of ERα and ERβ in Castration-Resistant Prostate Cancer and Current Therapeutic Approaches

**DOI:** 10.3390/biomedicines11030826

**Published:** 2023-03-09

**Authors:** Nur Erysha Sabrina Jefferi, Asma’ ‘Afifah Shamhari, Nur Khayrin Zulaikha Noor Azhar, Joyce Goh Yi Shin, Nur Annisa Mohd Kharir, Muhammad Afiq Azhar, Zariyantey Abd Hamid, Siti Balkis Budin, Izatus Shima Taib

**Affiliations:** 1 Center of Diagnostics, Therapeutics and Investigative Studies (CODTIS), Faculty of Health Sciences, Universiti Kebangsaan Malaysia, Jalan Raja Muda Abdul Aziz, Kuala Lumpur 50300, Malaysia; 2 Biomedical Science Programme, Faculty of Health Sciences, Universiti Kebangsaan Malaysia, Jalan Raja Muda Abdul Aziz, Kuala Lumpur 50300, Malaysia

**Keywords:** castration-resistant prostate cancer, estrogen receptor, anti-estrogen therapy, antioxidant, immunotherapy

## Abstract

Castration-resistant prostate cancer, or CRPC, is an aggressive stage of prostate cancer (PCa) in which PCa cells invade nearby or other parts of the body. When a patient with PCa goes through androgen deprivation therapy (ADT) and the cancer comes back or worsens, this is called CRPC. Instead of androgen-dependent signalling, recent studies show the involvement of the estrogen pathway through the regulation of estrogen receptor alpha (ERα) and estrogen receptor beta (ERβ) in CRPC development. Reduced levels of testosterone due to ADT lead to low ERβ functionality in inhibiting the proliferation of PCa cells. Additionally, ERα, which possesses androgen independence, continues to promote the proliferation of PCa cells. The functions of ERα and ERβ in controlling PCa progression have been studied, but further research is needed to elucidate their roles in promoting CRPC. Finding new ways to treat the disease and stop it from becoming worse will require a clear understanding of the molecular processes that can lead to CRPC. The current review summarizes the underlying processes involving ERα and ERβ in developing CRPC, including castration-resistant mechanisms after ADT and available medication modification in mitigating CRPC progression, with the goal of directing future research and treatment.

## 1. Introduction

Prostate cancer (PCa) is the second most prevalent malignancy and the fifth most common factor in cancer-related deaths, with a 13.5% incidence rate and a 6.7% mortality rate among men worldwide [1,2]. Prostatic tumours, both benign and malignant, are uncommon among men before the age of 40 and are more prevalent with age. PCa is an androgen-dependent cancer. The majority of PCa cases can be detected at the stage of adenocarcinoma; the signs and symptoms are undetectable at earlier stages. Locally advanced adenocarcinoma has resulted in the implementation of androgen deprivation therapy (ADT) in conjunction with other therapeutic modalities, such as radiotherapy and radical prostatectomy [3]. Since 1972, ADT has been the gold standard of care for the first-line management of advanced or metastatic prostate cancer. ADT may involve luteinizing hormone (LH) agonists, LH antagonists, or orchiectomy, and the main target of this therapy is to reduce testosterone action. Some patients respond to ADT treatment and show a relapse of cancer cells. However, within 2–3 years following the start of ADT, some patients may develop castration-resistant prostate cancer (CRPC), the prognosis for which is worse than ADT [4].

Several pathways are involved in the development of CRPC: (i) androgen receptor (AR) amplification and hypersensitivity, (ii) promiscuity-causing AR mutations, (iii) mutations in coactivators, and (iv) intratumoural and alternative androgen production. CRPC is a state of PCa that is, to date, incurable [5,6]. If CRPC is left untreated, the cancer can progress to metastatic CRPC (mCRPC). Further, CRPC progression has been found to be associated with estrogen regulation through the mediation of nuclear receptors, namely estrogen receptor alpha (ERα), which is an androgen-independent receptor, and estrogen receptor beta (ERβ), which is an androgen-dependent receptor [7,8]. These receptors have contradictory functions: ERα promotes proliferation, whereas ERβ possesses antiproliferative properties in prostate epithelial cells. The expression of ERα in prostate stromal cells varies during neonatal development, and no ERα can be detected by week 4. During the early development of the prostate, these ERα expressions are necessary to mediate the production of stromal factors and stimulate the growth and differentiation of epithelial cells [9]. After ADT, low androgen levels reduce the functionality of ERβ in suppressing tumour progression. Meanwhile, ERα continues to excessively promote the proliferation of PCa cells, resulting in the invasion and migration of PCa cells in blood circulation [10].

The molecular networks of estrogenic signalling via ERα and ERβ in PCa have not yet been fully understood. The significant functions and underlying mechanisms of ERα and ERβ in the development of CRPC remain unclear. However, clinical scientists have found that ERα and ERβ have the potential to be clinically targeted molecules when treating PCa at the CRPC stage. Numerous clinical trials on the molecular and functional aspects of antiestrogen treatment for PCa have been carried out. The usage of selective estrogen receptor modulators (SERMs) and antioxidants, whose effectiveness has been thoroughly evaluated in both preclinical and clinical models of CRPC, has paved the way for cutting-edge therapeutic approaches for the treatment of PCa [11,12]. The present review focuses on the roles of ERα and ERβ in CRPC and the application of therapeutic modulations that target these two receptors, with the aim of elucidating the treatment alternatives for attenuating CRPC.

## 2. Role of ADT in Developing CRPC

ADT, specifically surgical or medical castration, is the standard first-line treatment for high-risk PCa, which includes locally advanced adenocarcinoma as well as PCa with advanced metastatic PCa. ADT is beneficial for palliation in patients with advanced PCa and improves the prognosis of high-risk patients treated with radiotherapy for PCa. ADT is known for putting diseases into remission, which has been shown by a drop in prostate-specific antigen (PSA) in about 90% of patients [13]. However, after an average of 2–3 years of ADT treatment, ongoing hormonal manipulation causes CRPC to develop. CRPC is an aggressive type of PCa that affects 10–20% of patients who undergo ADT treatment [14]. It is well known that testosterone plays a role in fostering the development of PCa; therefore, inhibiting or reducing its production can reduce the growth of malignant cells in the prostate [15]. ADT approaches are of different types, involving orchiectomy, luteinizing hormone-releasing hormone (LHRH) agonists, or LHRH antagonists.

Approximately 90% of testosterone in the body is produced by the testes. Therefore, orchiectomy, the surgical removal of one or both testes, is a highly efficient method for reducing testosterone synthesis, and it can reduce blood testosterone levels by about 90–95% [16]. This strategy has been utilized effectively since the 1940s. However, since it is permanent and irreversible, most men prefer drug therapy instead of orchiectomy. Orchiectomy is typically performed as an outpatient procedure in the urologist’s clinic. Patient recovery is typically rapid, and no additional hormone medication is required, making it a promising option for those who prefer a low-cost, one-time procedure. Additionally, it is associated with a low incidence of cardiovascular problems and fractures compared to hormone replacement treatment [17].

LHRH agonist drugs, sometimes referred to as LHRH analogues (also known as gonadotropin-releasing hormone (GnRH) agonists or GnRH analogues), are structurally identical synthetic proteins that bind to the LHRH receptor in the pituitary gland. When androgen levels are low, the hypothalamus often releases LHRH. This stimulates the testes to synthesize androgens. LHRH agonist drugs are similar to the body’s endogenous LHRH; they stimulate LH synthesis from the pituitary gland. However, at high doses, the long-term, persistent stimulation of LHRH agonist drugs causes the pituitary gland to stop producing LH [18]. Therefore, the testes do not receive any stimulation to produce androgen, causing the androgen level in the blood to decrease further. Castration with an LHRH agonist drug is known as a medical or chemical castration. In contrast to orchiectomy, the effects of these medications on androgen production are reversible, and androgen production resumes once the medication is discontinued. LHRH agonist drugs are administered via injection or skin implantation. In the United States, four LHRH agonists are licensed for the treatment of PCa, namely leuprolide (Lupron), goserelin (Zoladex), triptorelin (Telstar), and histrelin (Vantas). When patients receive an LHRH agonist drug for the first time, they may suffer from a phenomenon known as a testosterone flare [19], which is a transitory increase in testosterone levels due to the LHRH agonist drugs causing the pituitary gland to secrete additional LH for a short period. At some point, the pituitary gland starts inhibiting the release of LH. During the first few weeks of treatment, antiandrogen drugs are usually administered along with the LHRH agonist to counteract the rise in androgen. Flutamide, enzalutamide, and apalutamide are examples of antiandrogens that are used in conjunction with ADT [3].

Another option for ADT is the use of LHRH antagonist drugs. LHRH antagonists (also known as GnRH antagonists) prevent LHRH from binding to its receptors in the pituitary gland. This suppresses the release of LH, which reduces the androgen synthesised by the testes. Unlike LHRH agonist drugs, LHRH antagonist drugs do not elevate testosterone levels [20]. Two LHRH antagonist drugs have been approved for the treatment of advanced PCa in the United States: degarelix (Firmagon) and relugolix (Orgovyx). These medications have different, rapid mechanisms of action for reducing patients’ testosterone levels, and they do not trigger testosterone flares. In sum, this is a relatively new class of medications that can block LHRH (GnRH) from acting on the pituitary gland and releasing LH for testosterone production without causing an initial rise in testosterone levels.

ADT is normally applied to those who are diagnosed with locally advanced adenocarcinoma [21]. For many years, ADT alone was the conventional treatment for treating PCa in its advanced stages [22]. Recent clinical trials have shown that patients treated with ADT along with another type of hormone therapy (abiraterone/prednisone, enzalutamide, or apalutamide) live longer than those treated with ADT alone [23,24,25]. In addition, a study has shown that male patients with hormone-sensitive metastatic PCa live longer when treated with the chemotherapeutic drug docetaxel (Taxotere) at the onset of ADT than patients treated with ADT alone [26]. Although hormone therapy can halt disease progression and may extend survival, it can also have significant, systemic negative effects, such as lowered libido, erectile dysfunction, bone fractures, and loss of bone density [27,28].

It is not possible for clinicians to estimate the duration of hormone therapy’s effectiveness in halting the progression of PCa in a patient. Therefore, it is mandatory for patients who have undergone ADT for more than a few months to have their PSA levels assessed. An increase in PSA levels may be a sign that a patient’s cancer has recurred. A PSA score that continues to rise despite the efficacy of hormone therapy in maintaining extremely low androgen levels indicates that the PCa has developed resistance to the administered ADT. This condition characterizes CRPC, which can be evaluated by conducting radiological and biochemical analyses [29,30] of PSA levels. The radiological assessment of PCa progression is guided by Response Evaluation Criteria in Solid Tumors (RECIST). The current imaging modalities proposed by the RECIST guidelines [31] are used to assess the progression of bone metastases when more than two hot spots appear. Meanwhile, PSA levels may increase three times in a row within one week, with at least two of these increases being more than 50% of the lowest levels of PSA. Together, increased PSA and bone metastases are indicative of PCa progression following ADT treatments, leading to CRPC [30].

Initially, the function of ADT is to induce senescent growth arrest, which results in tumour shrinkage. Senescence is considered a positive consequence of ADT because it has the potential to activate the immune system through the senescence-associated secretory phenotype and slow down or eliminate PCa progression. However, there is increasing evidence that senescent cells can regain their proliferative potential and that cancer may reoccur. Carpenter et al. [32] proposed that ADT escapes its function via a variety of mechanisms, including senescence escape, cell-autonomous reprogramming, and the formation of a protumourigenic senescence-associated secretory phenotype (SASP). These mechanisms have the potential to promote CRPC development via ADT-induced senescence.

The majority of mechanisms linked to CRPC have been identified as being mediated by AR. CRPC may develop via AR amplification and hypersensitivity. Following ADT, androgen levels remain low, and this microenvironment causes a subpopulation of prostate cells to develop sensitivity to the low amounts of androgen through the amplification of the AR gene. This hypersensitivity pathway promotes disease progression [31]. The development of CRPC may also be due to AR mutation; most of the cases in this regard have been reported among African-American patients. The mutation was detected in the leucine substitution of valine at codon 89. A large number of AR mutations can trigger the binding of adrenal androgens and other steroid hormones to AR. Mutations in AR coregulators or coactivators also characterize one of the possible mechanisms of CRPC development. Mutations in AR coactivators may occur in patients who undergo ADT. The functions of coactivators include enhancing the transcription activity of AR [33]. Therefore, mutated AR coactivators stimulate the transcription activity of AR even at extremely low concentrations of testosterone, resulting in the excessive proliferation of PCa.

In addition to ADT-induced senescence and AR-mediated CRPC, alternative pathways of steroidogenesis and estrogen signalling can cause the development of CRPC [34,35,36]. Dehydroepiandrosterone (DHEA) and its sulphated derivative (DHEA-S) are the precursors of androgen production in the adrenal cortex. These two molecules transform into the highly potent dihydrotestosterone (DHT) via a “backdoor” mechanism. DHT is the most potent androgen, and it is 2.5–3.0 times more potent than testosterone. Therefore, prostate cells can utilize DHT to proliferate the adenocarcinoma of PCa into CRPC. Estrogen signalling pathways mediated by the nuclear receptors ERα and ERβ have also been found to contribute to the progression of CRPC. The increased expression of ERα and the genes related to ERα observed during the progression of CRPC indicate that ER signalling can bypass AR for tumour growth following ADT [11]. Despite the AR’s involvement in the development of CRPC mechanisms, this new finding demonstrates that the role of ER signalling in the CRPC mechanism could provide further insights for addressing untreated PCa in men.

## 3. Roles of ERα and ERβ in Castration-Resistant Prostate Cancer

Several studies have assessed the role of ER in CRPC by utilizing three primary prostate cancer cell lines: PC-3, LNCaP, and DU145 [37]. Although cell lines are widely used in PCa research, evaluating the role of ERα and ERβ in these models is essential to determine whether these hormone receptors are involved in the development of CRPC. Due to resistance to ADT, intratumour androgen synthesis has been found to be elevated in CRPC. Simultaneously, E2 synthesis increases due to the activation of aromatase enzyme activity, indicating that a close relationship exists between these two steroid hormones. As the disease progresses, the alternative promoters of cytochrome P450 19A1 (CYP19A1) (a gene that encodes aromatase), such as the 1.3 and 1.4 promoters, are activated; these are initially inactive in normal prostate epithelial cells [38]. The activation of alternative promoters eventually leads to the increased expression of aromatase. It is anticipated that enhanced aromatase enzyme expression will result in higher E2 levels in tumour cells, which are, in turn, associated with greater aggressiveness of the disease. To the best of our knowledge, the roles of ERα and ERβ in PCa are still unclear because very few of their target genes have been identified thus far.

E2 is involved in the development of PCa via the expression of both ERα and ERβ in the prostate [39]. Similar to AR, E2 binds to and activates either ERα or ERβ. The activated receptor then dimerizes to form a complex homodimer or heterodimer and eventually initiates the relocalization of this receptor to the nucleus. In the nucleus, this complex binds to specific genomic deoxyribonucleic acid (DNA) motifs known as estrogen response elements (EREs). In addition, similar to AR, ERs quickly initiate nongenomic functions by adhering to the cell membrane and activating a series of signalling cascades [40,41].

Increased ERα has been found in PCa cells and is thought to contribute to cellular proliferation and inflammation, suggesting that this receptor has the potential to mediate the estrogenic activity that occurs during PCa onset and development [7]. The binding of a homodimer or heterodimer complex of ER to the ERE promotes matrix metalloproteinase 12 (MMP12), leading to an increase in PCa metastasis [42] and the overexpression of aromatase. Previous research has reported that MMP12 promotes cancer invasion during the interaction between PCa cells (i.e., PC-3) and bone marrow stromal (BMS) cells [43]. The interaction between PC-3 and BMS cells mimics the metastasis in PCa. Moreover, MMP12 has also been found to be involved in increasing CD44+ PCa cells (prostate cancer stem-like cells (PCSLC) associated with chemoresistance), leading to the overexpression of aromatase and increased intracellular E2 in CRPC [43]. Therefore, along with the presence of MMP12, ERα might play an important role in the development and progression of CRPC [44,45,46].

**Figure 1:**
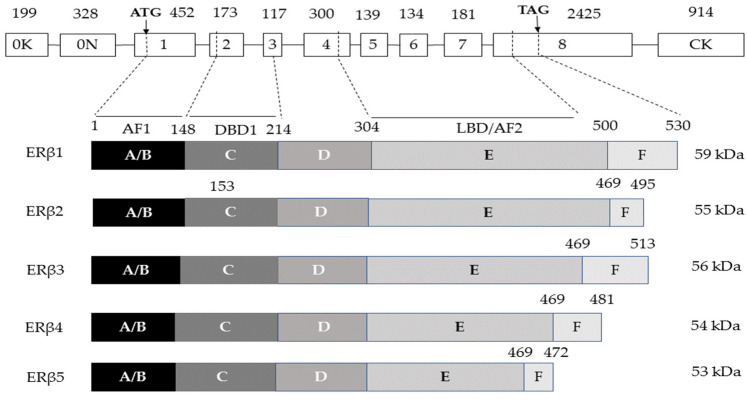
ESR2 gene and its five protein isoforms are represented schematically. Exons 1–8 are depicted by boxes, exons 0K and 0N are two promoters at the gene’s 5′ end, and introns are represented by lines. Numbers above the boxes indicate the size (bp) of each exon, arrows point to the start (ATG) and stop (TAG) codons, and dotted lines connect the gene regions to the protein domains or isoforms that are encoded, from N-terminus to C-terminus, A/B: activation function 1 (AF1) domain, C: DNA-binding domain (DBD), D: hinge domain, E: ligand-binding domain (LBD) or activation function 2 (AF2) domain, F: C-terminal domain. The molecular weight of each isoform in kDa is shown by the numbers on the right side. The figure is adapted from [54].

ERβ has the potential to mitigate PCa by restricting androgen-induced proliferation [42]. ERβ has five different isomer types: ERβ1, ERβ2, ERβ3, ERβ4, and ERβ5. The expression levels of ERβ isomers vary, as they are expressed differentially during the progression of PCa. ERβ1, ERβ2, ERβ4, and ERβ5 are expressed in the prostate [47,48,49]. ERβ2 and ERβ5 are regularly located in the cytoplasm, while ERβ1 is primarily found in the nucleus of prostate cells. ERβ5 has been found to mediate PCa progression; however, the involvement of this isomer in CRPC has not been clearly reported [47]. ERβ genes (i.e., ESR2) produce proteins with 530 amino acids which are localized on chromosome 14. Similar to ERα, eight exons are found in the ERβ gene [50,51]. All ERβ isoforms share exons 1–7, but exon 8 is unique to each isoform [52,53]. Exon 8 is alternatively spliced in the isoforms, generating proteins that have less molecular weight than the wild-type protein. Figure 1 shows a schematic representation of the ERβ isomers.

A study by Mak et al. [55] showed that ERβ1 and its specific ligand, 5α-androstane-3β,17β-diol (3β-Adiol), suppress the mesenchymal characteristics of prostate carcinoma. ERβ causes the destabilization of hypoxia-inducible factor 1-alpha (HIF-α) and represses the transcriptional activity of vascular endothelial growth factor A (VEGF-A) and transforming growth factor beta (TGF-β) in both PC-3 and LNCaP cell lines [55]. Furthermore, it has been established that ERβ expression decreases during the progression of PCa to CRPC [56]. The reduction of ERβ1 expression results in a significant increase in the migration and invasion of PCa cells, leading to the progression of the epithelial-mesenchymal transition (EMT). EMT is a process that can develop during the advancement of PCa tissues and is characterized by morphological changes from cuboidal to spindle-shaped in the phenotype [44].

Even though ERβ has antiproliferative, anti-invasive, and proapoptotic activities [57,58], this ER type is also involved in mediating the proliferation, migration, and invasion of PCa cells [59]. The activation of ERβ in PCa cells is mediated by WNT/beta(β)-catenin pathways [35] and increases in PC-3 cell lines, leading to the increased expression of non-phosphorylated β-catenin. This event causes the formation of the ERβ-β-catenin-TCF/LEF complex, which is required for PC-3 cell proliferation, migration, and invasion. Furthermore, the Wnt signalling pathway supports the β-catenin pathways through the Wnt secretion mediator Wntless (WLS) in CRPC in the enzalutamide-resistant cell line model C4-2B [59]. This shows that WLS might be activated via the ERβ-β-catenin-TCF/LEF complex and potentiate resistance to enzalutamide, a type of next-generation antiandrogen therapy (NGAT), leading to the development of CRPC.

Despite the involvement of WNT/beta(β)-catenin pathways, the mRNA expression of cyclin D1 (CCND1), a G1 cyclin, has been found to be involved in the development of castration-stage PCa. CCND1 is amplified and overexpressed in a variety of human malignancies, including PCa [60]. Furthermore, dysregulated CCND1 expression drives abnormal G1 to S transitions in the cell cycle, resulting in uncontrollable cell growth and carcinogenesis [61,62]. According to a study by Nakamura et al. [63], the expression of the CCND1 mRNA is enhanced after 48 h of treatment with E2 (10 nM) in PC-3 cells. In the same study, the researchers found that E2 promotes CCND1 expression via ERβ through the elevated expression of Fos and Jun in PC-3 cells. Thus, ERβ induces PCa occurrence and further progression to CRPC.

Steroid receptor coactivator/phosphoinositide 3-kinase/AKT/mammalian target of rapamycin (SRC-PI3K-AKT-mTOR) is another ER-mediated signalling pathway involved in CRPC progression. Both ERα and ERβ are associated with the phosphorylation of steroid receptor coactivator (SRC). A study by Lombardi et al. [35] demonstrated that ERα and ERβ activation via the ERα-selective agonist propylpyrazoletriol (PPT) and the ERβ-selective agonist diarylprepionitrile (DPN), respectively, causes an increase in the phosphorylation of SRC in the PC-3 and DU145 CRPC cell lines. SRC is a nonreceptor tyrosine kinase that is highly expressed in several PCa cell lines as well as in the majority of tissues derived from PCa [64]. SRC is induced by various cellular signalling molecules and has a significant effect in regulating numerous processes in signalling pathways, including cell growth, differentiation, adhesion, and migration [65]. Meanwhile, treatment of PC-3 cells with the P13K selective inhibitor Wortmannin and the AKT inhibitor MK2206 can prevent cell invasion induced by DPN (100%) or PPT (80%). Research findings indicate that ERα-PI3K/AKT and ERβ-PI3K/AKT are involved in the invasion capability of PC-3 cells [35].

Most of the previous studies on the roles of ERα and ERβ have utilized PCa cell lines. However, during cell culture, immortalised cells have the potential to dedifferentiate and experience genetic or phenotypic modifications. This results in the loss of expression of hormone receptors such as AR and ERs [66,67,68]. Although PCa cell lines are simple to employ, reproducible, and cost-effective, their main drawbacks are genetic shifts and the acquisition of mutations driven by prolonged culture periods. In a study by Barbieri et al. [69], chromodomain-helicase DNA-binding protein 1 (CHD1) deletion and forkhead box protein (FOX) A1 and speckle-type POZ protein (SPOP) mutations were not detected in PCa cell lines, although these genetic changes were detected in PCa patients. Given these discoveries, the application of small mammalian animal models in cancer studies has become a promising alternative for assessing CRPC due to the accessibility of numerous mouse strains with specific gene modifications [70]. A number of in vivo tumour propagation models, such as genetically engineered mouse (GEM) models, patient-derived xenograft (PDX) models, and 3D cultures of patient-derived PC cells (organoid or sphere cultures), have been established by researchers to circumvent the restrictions of PCa cell lines [71].

GEM models include transgene-expressing and knockout mice, such as transgenic adenocarcinoma of the mouse prostate (TRAMP), LADY, SV40, Cre-recombinases, phosphatase and tensin homolog (PTEN), and Myc models. To the best of our knowledge, only one study has investigated the role of ERs in PCa using a GEM model. Slusarz et al. [72] found that ERα knocked-out (KO) TRAMP mice had essentially no incidence of poorly differentiated carcinoma (PDC); however, ERβKO mice had roughly double the incidence of PDC (39%) compared to wild-type mice. This is consistent with prior claims that ERα increases and ERβ lowers the risk of PCa [73]. Therefore, targeting the ERs might help prevent the progression of PCa to CRPC.

Human prostate cancer tissue obtained from prostate or metastatic locations is used to create patient-derived models. Cancer tissues are harvested from these sites and subsequently processed for patient-derived xenografts (PDXs), patient-derived explants (PDEs), and patient-derived organoids (PDOs) grown in three-dimensional (3D) cultures with dissociated cells. Organoids are small, organ-like structures that possess crucial organ characteristics and are commonly used as transitional models between PDXs and in vitro cancer cell lines. Organoids are capable of self-renewal and self-organization and can efficiently and accurately mimic the in vivo microenvironment as well as the molecular and genetic signatures of the tissues or organs of origin [74,75]. Notably, the expression and activation of hormone receptors, such as ERα and ERβ, are preserved in mammary gland organoids [76]. The preservation of these ERs can facilitate their evaluation in organoid models. However, to the best of our knowledge, no studies have focused on the roles of ERα and ERβ in PCa organoids. Therefore, it is necessary to use organoid models to evaluate how these receptors influence PCa development and progression. In addition to employing fresh primary specimens, PDX-derived tissues can be used for PDE and PDO studies. This enables high-throughput analyses of long-term propagating tumours, thus increasing the utility of the models. Further, PDOs can be transplanted back into mice to develop as PDXs, enabling their long-term development in vivo [77].

Several CRPC PDXs from patients receiving modern medicines or those who underwent numerous lines of treatment have been produced [78,79,80,81]. In addition, previous studies utilizing PDXs to evaluate ER only assessed breast cancers [82,83,84]. Research on the role of ER in PCa, especially in CRPC, using PDXs is not yet available. Most of the existing studies involving CPRC PDXs have demonstrated the involvement of AR signalling pathways in CRPC progression. A study by Hemelryk et al. [81] detected AR signalling in PDXs and matching PDX-derived organoids (PDXOs) in four PDXs of CRPC patients with AR amplification. Further scientific exploration of CRPC via ER signalling pathways using PDX and 3D cultures of patient-derived PC cell models is needed to enhance preclinical testing methods and thus establish improved treatments for patients with PCa.

Clinical studies involving cohorts of CRPC patients have assessed the functional role of ER by utilizing human data. In a study by Qu et al. [45], four estrogen receptor 1 (ESR1) mutants (E380Q, L536Q, Y537S, and D538G) were detected in ribonucleic acid (RNA) samples from PCa patients whose initial blood samples were taken prior to their becoming castration-resistant. This finding revealed that castration resistance is linked to the presence of ERα mutations. Furthermore, Qu et al. [45] also demonstrated that the concentrations of ERβ splice variants declined following additional successive treatments, indicating the loss of ERβ. A large cohort study that examined the primary tumour tissue of 535 patients who had a radical prostatectomy (RP) revealed that low expression levels of ERβ are linked to low chances of avoiding biochemical failure, confirming that a reduced level of ERβ leads to CRPC [46].

In a study by Zellweger et al. [57], a significant increase in ERβ1 expression was demonstrated in a CRPC patient cohort following the development of castration resistance. In contrast, ERα was only found in 9% of the CRPC samples. ERβ isomers, namely ERβ2 and ERβ5, were strongly associated with prostate cancer metastasis, suggesting that they possess oncogenic properties in PCa patients. PCa cell invasion was increased by both ERβ2 and ERβ5, but only those cells expressing ERβ5 showed an increase in cell migration [47]. The prevalence of ESR1 (which encodes ERα) and ESR2 (which encodes ERβ) mutations was 3% (5/150) in individuals with metastatic or advanced PCa, whereas it was 2% (11/492) in patients with early prostate cancer, according to a genomic dataset [63].

**Figure 2:**
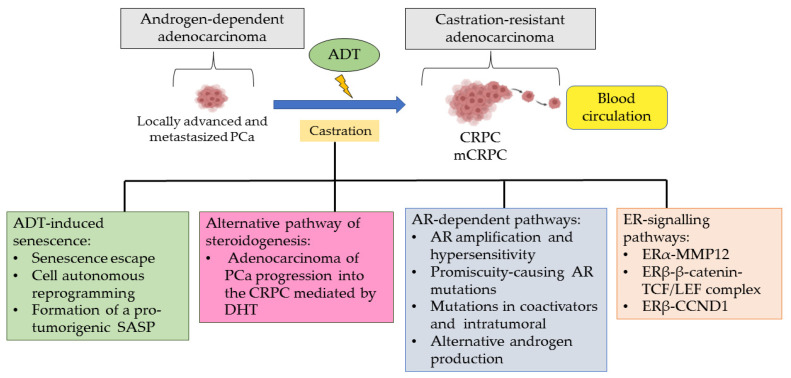
PCa development leading to CRPC progression after ADT treatment via AR-dependent alternative pathway of steroidogenesis and an ER-signalling pathway. The possible mechanisms of PCa leading to CRPC after ADT treatment could be due to the androgen-dependent pathway, including AR amplification and hypersensitivity, AR mutation, and AR co-regulation (co-activation). Next, the alternative pathway of steroidogenesis will also be the potential mechanism of PCa progression to CRPC. ADT-induced senescence, including senescence escape, cell-autonomous reprogramming, and the formation of a pro-tumorigenic SASP. Abbreviations: PCa, prostate cancer; ADT, androgen-deprivation therapy; AR, androgen receptor; SASP, senescence-associated secretory phenotype; CRPC, castration-resistant prostate cancer; mCRPC, metastatic castration-resistant prostate cancer; ERα, estrogen receptor alpha; Erβ, estrogen receptor beta; MMP12, matrix metalloproteinase 12; TCF/LEF, T cell factor/lymphoid enhancer factor transcription factors; β-catenin, beta-catenin; CCND1, cyclin D1.

Although previous studies have reported that ERβ isomers play different roles in regulating PCa progression, research on the involvement of these isomers in CRPC is still scarce. Most studies have reported on the role of ERβ in CRPC without evaluating the types of isomers involved. Therefore, the dual roles of ERβ in regulating CPRC progression, either increasing or repressing the progression of prostate tumours, differs according to the distribution of various ERβ isomers in PCa tissues. The question of which ER mediates the impact of estrogen on prostatic growth and differentiation at the castration stage has direct significance for understanding the risk of recurrent prostatic disease. These findings are also beneficial for the development of pharmaceuticals for the treatment of CRPC. Table 1 provides a summary of the castration-resistant prostate cancer study models used to evaluate ERα and ERβ expression. Figure 2 shows how PCa development leads to CRPC progression after ADT, mediated by ERα and ERβ via alternative estrogen signalling pathways.

## 4. Current CRPC Treatment and Challenges

The tumour microenvironment (TME) has a significant impact on cancer cell survival and the development of treatment resistance [86]. The majority of solid tumours are comprised of tumour cells combined with noncancerous cells, which are sustained by a disordered vascular network; this results in hypoxia, decreased nutrition delivery, and the inefficient clearance of metabolic breakdown products. Erratic blood flow also restricts drug delivery in blood circulation. In conjunction with the hypoxic state, the TGF, fibroblast growth factor (FGF), β-catenin, and mTOR pathways play a role in the epithelial–mesenchymal transition (EMT). These underlying mechanisms have been associated with the development of metastases as well as with increased invasiveness and resistance to chemotherapy in CRPC cases [87].

Radiotherapy is another common type of PCa treatment that is applied to generate DNA base breaks, single-strand breaks (SSBs), and double-strand breaks (DSBs) in PCa cells [88]. Wang et al. [89] discovered that DNA damage in DU145 cells was fully repaired 24 h after radiotherapy (or 48 h in PC-3 cells). The researchers proposed that the loss of NEIL3, a DNA glycosylase family member that initiates the first stage of base excision repair via an associated lyase reaction, triggers radiotherapy resistance via the ATR-CHK1 DNA damage response (DDR) pathway. Resistance to NGATs, such as enzalutamide, apalutamide, or darolutamide, has also been reported [90]. Overexpression of AR, mutations in the AR ligand-binding domain (LBD), deletion of AR, constitutively active AR splice variants (AR-SVs), increased intratumoural hormone production, and the activation of growth factor pathways have all been linked to resistance towards NGATs. AR splice variants encode a modified form of AR protein with a missing C-terminal LBD but a functionally intact N-terminal domain, a partial or complete DBD, and the ability to interact with DNA and AR coreceptors. The fact that AR splice variants lack an LBD and that both enzalutamide and abiraterone are activated by binding to the LBD suggests a potential mechanism of resistance [91].

Immunotherapy has been utilized in treating CRPC via the usage of cellular vaccines and immune checkpoint inhibitors (ICIs). Unfortunately, prospective immune resistance mechanisms lead to a low likelihood of clinical efficacy in prostate cancer cases [92]. Prostate cancers exhibit low immunogenicity, as indicated by a low mutational burden, decreased neoantigen generation, decreased PD-L1 expression, and reduced HLA-1 molecule expression [93]. Moreover, a significant body of evidence indicates that the prostate TME contains immunological suppressive cells, such as regulatory T cells, regulatory B cells, and myeloid-derived cells, which inhibit T cell invasion and clonal proliferation [94]. These mechanisms induce cancer progression and the development of castration resistance in CRPC patients [94,95].

## 5. Molecular Targeted Therapeutic Approaches in Mitigating CRPC

### 5.1. Drugs Targeting Estrogen Related to CRPC Treatment

Patients with CRPC used to have a low chance of survival, but treatments for this disease have come a long way in recent years [16]. Treatment options beyond cytotoxic chemotherapy are now available. These include a number of new drugs that not only target androgen- and cytotoxic-mediated pathways but also affect the patient’s immune system [60]. Preclinical studies have been conducted, and clinical trial endpoints such as PSA levels, overall survival (OS), and progression-free survival have been used to compare and evaluate new CRPC treatments. Many drugs are being developed for the treatment of CRPC, either alone or in combination with other drugs. Four drugs are now commonly used to treat CRPC: docetaxel, prednisone, cabazitaxel, abiraterone acetate (AA), and sipuleucel-T. However, these drugs need to be administered together to be effective and improve the prognosis [60]. The first-line treatment choices currently available include docetaxel and sipuleucel-T [96]. If the first-line treatment does not work, the second-line medicines can be used, such as cabazitaxel and AA.

Docetaxel, prednisone, and cabazitaxel are potent inhibitors of microtubule depolymerization. The activity of cabazitaxel is resistant to P glycoprotein, the adenosine triphosphate (ATP)-dependent drug efflux pump that is sometimes expressed in cancer cells [97]. Meanwhile, AA is a highly selective, potent inhibitor of the cytochrome P450 17A1 (CYP17A1) enzyme, which leads to a decrease in the proliferation of PCa cells [85]. Sipuleucel-T is a type of immunotherapy that has been approved for any cancer cell; it targets AR and causes cell cycle arrest [98]. This approach has been designed to stimulate T cell immunity against prostatic acid phosphatase, a type of antigen that is expressed on the surface of PCa tumour cells. Notably, all of the listed drugs specifically target AR, leading to cell cycle arrest and inducing the immune system; however, the combination of docetaxel and prednisone together with ADT has been found to be active towards ERs [99]. In a past study, ADT (LHRH analogues), docetaxel (75 mg/m^2^), and prednisone (10 mg/day) were administered to 125 metastatic hormone-sensitive PCa (mHSPC) patients every 21 days for six cycles. These drug combinations resulted in low ESR1 and high ESR2, with no relationship between ESR and clinical factors; the combinations were independently associated with better outcomes and longer CRPC durations. The researchers also reported an inverse correlation between ESR2, AR, and ARV7 (oncogenic drivers in CRPC) and a proportional correlation with PTEN involved in the apoptosis of PCa cells [100].

Another treatment option is hormone therapy. Hormone therapy is a powerful and safe treatment that helps hormone receptor-positive tumours perform better clinically [101]. Evaluations have been carried out to determine how hormone therapy can be used in more advanced ways as well as in adjuvant and neoadjuvant settings. Researchers have looked into different drug combinations and schedules to lessen the adverse effects of the therapy. Smith et al. [101] found that transdermal E2 at a dose of 50 mcg/24 h resulted in a >50% decrease in PSA in 5 out of 40 steroid-resistant PCa patients (13%) after one month of treatment. The researchers stated that E2 stopped the PCa cells from growing by changing the function of the sex hormone-binding globulin through what is called an ER-independent pathway. Apart from the hormone itself, hormone metabolites have a significant effect on tumour cell growth in CRPC by targeting ERβ. A previous study found that 3β-Adiol and 3α-Adiol, as well as DHT and R1881 (an anabolic–androgenic steroid), can significantly reduce tumour cell growth in CRPC cells. These metabolites suppress tumour cell growth by increasing the expression of ERβ, which regulates estrogen signalling and mediates the antiproliferation effects [102].

### 5.2. Anti-Estrogen Therapies

The most commonly used antiestrogen therapies are SERMs and selective estrogen receptor downregulators (SERDs). Both of these antiestrogen therapies represent a significant development in clinical therapy. SERMs have specific agonist or antagonist effects on a variety of target tissues with estrogen receptors, such as the breast, bone, prostate, and testes. Meanwhile, SERDs act as antagonists toward estrogen receptors. The chemical structure of SERMs is unique as they lack in the steroid structure of estrogen but contain a tertiary structure that enables them to bind with estrogen receptors, thus preventing ER signaling. Although SERDs have the ability to bind to ER and prevent ER signalling, this therapy may cause ER degradation [103].

The ability of SERMs to exert either an agonist or antagonist effect solely depends on its unique pharmacological interactive mechanism, which, in turn, depends on the expression of estrogen receptors in target tissues. Three stages of regulation—ER subtypes, ER dimer formation, and tissue-specific coregulator transcriptional proteins—can modify tissue responses to estrogen, making SERMs’ biological effects complex and challenging to anticipate or predict [102]. The target cells for estrogen action have varying amounts of homodimers of ERα and/or ERβ. Upon estrogen’s ligand–receptor binding, ER dimerization is activated, and ERs turn into ERα–ERα homodimers, ERα–ERβ heterodimers, and ERβ–ERβ homodimers [104]. The formation of these dimer combinations further modifies the tissue-specific responses of estrogen and SERMs in various tissues. Meanwhile, SERDs belong to a distinct class of medications that prevent ER activation by lowering ER protein expression levels [105]. Once SERDs have bound to ERs, they create unstable protein complexes and cause conformational changes that result in the proteasome degrading the ER proteins.

The actions of SERMs also depend on the expression of coregulatory proteins upon binding to the estrogen receptors. The activation of tissue coregulatory proteins further alters the tissue’s biological responses to SERMs, leading to agonist or antagonist actions toward the tissue [106]. More than 20 coregulator proteins have been identified so far; they bind to estrogen receptors and alter their functions by exhibiting positive or negative transcriptional regulators (via coactivators or corepressors, respectively). Generally, SERMs exhibit estrogenic agonist actions in a tissue-specific manner (prostate cancer, breast cancer); however, the drugs also exhibit antiestrogenic actions as antagonists towards other tissues (bone, uterus) throughout the body [56]. This dual function of SERMs may be due to the pharmacological actions of SERMs. Variable combinations of ligand binding between coregulator proteins interact with estrogen receptors’ unique receptor conformations. This modulates the functions of SERMs in a variety of ways, including their agonist or antagonist responses.

Protein crystallography and surface evaluation techniques have demonstrated that binding by different ligands, such as estradiol (E2), tamoxifen, raloxifene, or the pure estrogen antagonist ICI 164,384, results in a distinct estrogen–receptor conformation for each ligand. For instance, estrogen and 3β-Adiol are the ligands for ERβ in the breast and prostate, respectively [102]. Thus, estrogen–receptor conformations caused by different ligand bindings lead to diverse outcomes, such as agonist or antagonist responses of the target tissues. This suggests that treatment mechanisms involving SERMs would exhibit different selective effects depending on the targeted tissues. Furthermore, the cellular response to estrogen, whether agonistic or antagonistic, will depend on the relative expression levels of these two estrogen receptors. Therefore, different target tissues, such as prostate and breast cancer tissues, exhibit different expression levels of these two estrogen receptors.

SERMs have been suggested and investigated for their potential to treat CRPC. The most commonly used SERMs in treating CRPC are toremifene, raloxifene and fulvestrant [8,107]. The estrogenic activity of ERβ reverses the morphological changes from cuboidal to spindle-shaped in PCa cells, whereas SERMs block ERα in overcoming the therapeutic resistance in CRPC [11]. Previous research has shown that elevating the activity of ERβ with the ERβ-selective agonist 8-VE2 in castration-resistant vertebral cancer of the human prostate (VCaP) cells results in a time- and dose-dependent reduction of cell survival and an increase in apoptosis. Moreover, the expression of AR proteins reduces by 52%, while AR mRNA is downregulated by 40% in VCaP cells. These findings indicate that ERβ activation using an agonist serves as an antiandrogen treatment for preventing CRPC progression [107].

In a translational pilot phase II clinical study by Shazer et al. [108], raloxifene was found to display tumour-inhibiting actions against CRPC. Raloxifene suppressed androgen-independent PCa development in 5 out of 13 patients (28%). In another study, 18 men with CRPC were administered a combination of raloxifene (60 mg) and bicalutamide (50 mg) across 28-day cycles to evaluate the safety of the dose for treatment. However, the advantages of the treatment were minimal, despite the fact that none of the patients required dose reduction. Only 4 out of 18 patients had a PSA reduction of more than 50%, with a median progression-free survival (PFS) of 1.9 months (1.8–2.8 months) [107]. Moreover, in a clinical trial study at phase II, 300–600 mg/m^2^ of toremifene was administered to 15 patients with CRPC. This is a significantly high dose of toremifene for treating CRPC. The treatment was well-tolerated by the patients and had minimal toxicity, but no PSA or radiographic responses were observed [109]. Hariri et al. [110] discovered that administering toremifene (ERα antagonist) via nano-targeted delivery increases the effectiveness of its anticancer properties. The nanoformulated toremifene blocks the estrogen hormone from binding to ER, reducing the tumourigenicity of PCa.

Fulvestrant, an estrogen antagonist without agonistic action, has been clinically investigated [8,111]. Fulvestrant is an SERD, specifically for ERα, that has the unique ability to increase the rate of receptor turnover, thus limiting the amount available for further activation. This emphasizes the therapeutic importance of SERDs for ERα inhibition and elimination. In a study, CRPC patients (*n* = 20) were intramuscularly injected with 500 mg of fulvestrant on day 0, followed by 250 mg on days 14 and 28, and monthly thereafter [111]. The treatment outcomes showed that the median time to cancer progression was 4.3 months, while the median overall survival of the CRPC patients was 19.4 months. No patient demonstrated a PSA decline of more than 50% or any radiological improvements. Therefore, SERDs constitute a novel therapy for CRPC in combination with other drugs such as raloxifene and bicalutamide [107]. Moreover, both SERDs and SERMs can be developed as therapeutic drugs that are individually specified for CRPC patients in conjunction with specialized medicine. This is because the dosages used in this therapy influence the efficiency of SERMs in treating any cancer, especially CRPC. Research findings indicate that the efficacy of SERMs in treating CRPC is debatable. This may be because of the selectivity of SERMs and the fact that ERβ agonists are not specific to the ERβ isomer, leading to off-target effects. Therefore, the positive outcomes of this treatment cannot be identified. Furthermore, there are controversial reports on how well the CRPC models, which include genetically altered mice, xenografts, and cell culture models, mimic the important features of the human prostate. Traditional CRPC models were shown by Lafront et al. [37] to be ineffective for studying the estrogen signalling pathway, and it was proposed that this may be a factor affecting the CRPC treatment outcomes of SERMs. Table 2 presents a list of treatments involving the usage of SERMs to prevent CPRC.

### 5.3. Selective Inhibitors

Various treatments have been progressively improved to mitigate PCa. The majority of current PCa treatments depend on inhibiting androgen signalling (e.g., ADT), which is beneficial in the early stages of the disease. However, this treatment eventually results in CRPC, and no viable follow-up alternatives are available. Anticancer drugs specific to CRPC are still being developed, and some new small molecules have proven to be very effective against this disease. Although most of these molecules are effective on their own, their antitumour effects are often tested in combination with other standard PCa treatments that target the kinase signalling pathway. Due to the common modifications that occur in kinase signalling pathways following the development of CRPC [31,113], inhibiting these tyrosine kinases and their respective downstream components is a therapeutic approach for CRPC. Inhibitor drugs targeting the kinase signalling system, which involves receptor tyrosine kinases, SRC kinases, and survivin, have not been successful in treating CRPC as a single therapy; only 25% of patients studied in this regard showed a stable status, and none were cured [106,113].

Activation of the PI3K-AKT-mTOR signalling pathway, whether through PTEN loss or independent hyperactivation of downstream effectors, is linked to prostate cancer progression and castration-resistant growth [114]. Growth factor stimulation is followed by activation of the PI3K pathway. This initiates a signalling cascade that ultimately results in the phosphorylation of phosphoinositide-dependent kinase (PDK1) and mTOR complex 2 (mTORC2). Immunohistochemical studies revealed increased expression of phosphorylated mTOR (p-mTOR) in PCa patients, leading to elevated biochemical recurrence of PCa after RP [115,116]. Phosphorylated mTORC2 and phosphorylated PDK1 are essential in activating AKT. Cell survival, cell cycle progression, and proliferation are stimulated by AKT activation via downstream effectors such as mouse double minute 2, glycogen synthase kinase 3, Bcl-2-associated death promoter (BAD), and forehead box O (FOXO). Drugs that block PI3K and downstream targets, such as pan-PI3K inhibitors, isoform-specific PI3K inhibitors, AKT inhibitors, and mTOR inhibitors, have been discovered by the role of the PI3K pathway in the growth and progression of PCa. Nevertheless, pan-PI3K inhibitors and selective PI3K inhibitors have not shown any therapeutic efficacy and may have serious side effects [117,118]. A phase II trial with the pan-class I PI3K inhibitor BKM120 alone or in combination with enzalutamide failed to show any apparent efficacy when administered in men with mCRPC. Most patients stopped receiving treatment because of progression within two to six months, and the trial was abandoned at the first stage due to its futility. Furthermore, grade 3 negative effects such as lung infection, multi-organ failure, urinary tract blockage, disorientation, and seizure occurred in 43% of patients [117]. ISA-2011B is another newly discovered selective PIP5K1a inhibitor that specifically inhibits advanced PCa by reducing the activity of the PI3K/Akt, AR, and ERα pathways [119]. Semenas et al. [119] demonstrated that treating PC-3 cells with tamoxifen in combination with ISA-2011B could significantly decrease PC-3 cell counts. In addition, ERα expression was found to be significantly suppressed in tumours treated with tamoxifen, ISA-2011B, or tamoxifen and ISA-2011B compared to controls in a xenograft mouse model harbouring castration-resistant tumour PC-3.

The utilization of AKT inhibitors also leads to severe negative effects [120]. A type of AKT selective inhibitor, AZD5363, was tested in combination with enzalutamide in mCRPC patients during a phase I study. The criteria for a response (reduction in prostate-specific antigen > 50%, circulating tumour cell conversion, and/or radiologic response) were met by three of the thirteen patients evaluated. However, several patients experienced side effects such as a rash (20%) and hyperglycemia (26.7%) [121]. Early preclinical research on mTORC1 inhibition showed moderate effectiveness, but further trials failed to show the continuation of therapeutic effectiveness. In a phase II trial, nine patients with CPRC who had previously taken either enzalutamide or abiraterone were treated with 4 mg daily doses of the dual mTOR inhibitor MLN0128, and six individuals reported significant side effects, with dyspnea being the most frequent [122]. In a study involving the PI3K/mTOR inhibitor, men with mCRPC who were progressing on abiraterone completed a phase I/II randomised trial of enzalutamide (ENZ) with or without the PI3K/mTOR inhibitor LY3023414 (LY). However, LY + ENZ had to be discontinued due to adverse events (AE). Diarrhea, nausea, and weariness were frequent adverse reactions of these combined therapeutic modalities [123]. Therefore, the usage of selective inhibitors in regulating the SRC-PI3K-AKT-mTOR signalling pathway must be improvised efficiently to reduce the side effects induced by these drugs, especially for CRPC patients. Table 3 indicates several selective inhibitors used in treating CRPC.

### 5.4. Antioxidants

The efficiency of natural product-based research for developing new, physiologically active, innovative pharmaceuticals has been demonstrated [124,126]. The standard treatment for CRPC is docetaxel, an anticancer drug used in combination with ADT. Over time, CRPC patients develop resistance to docetaxel, which may contribute to patient mortality. Several in vitro studies have shown that natural products have high anticancer efficacy for a range of malignancies; thus, natural products and their derivatives have gained prominence in the field of cancer research [126,127]. Notably, natural products have minimal toxicity profiles and are well tolerated by cancer patients [126,127,128].

A previous study has demonstrated the potential use of lycopene in combination therapy for treating CRPC [129]. Lycopene treatment was found to improve the growth-inhibiting impact of docetaxel in DU145 cells. Furthermore, in a DU145 xenograft tumour model, the combination of docetaxel and lycopene increased the antitumour effectiveness by 38% compared to docetaxel alone. A clinical trial on the combined effects of lycopene and docetaxel in treating CRPC patients is required, as preclinical data suggest that lycopene supplementation increases the anticancer efficacy of docetaxel.

In CRPC cells, δ-tocotrienol promotes cytotoxicity and apoptosis. Research findings have shown that δ-tocotrienol causes a decrease in cell viability and exerts a cytotoxicity effect on both DU145 and PC-3 PCa cell lines via an MTT assay. δ-tocotrienol has also been reported to exhibit proapoptotic effects by stimulating the endoplasmic reticulum stress pathway in PC-3 cells. These findings show that the anticancer effect of δ-tocotrienol in CRPC cells involves apoptosis (specifically via the endoplasmic reticulum stress pathway) and the paraptosis pathway [130].

Another study revealed the potential of melatonin as an anticancer agent. Melatonin treatment was found to inhibit tumour growth and reverse enzalutamide resistance in animal CRPC models with a circadian rhythm disorder. Furthermore, the carboxylesterase 1 (CES1) gene, which is known to stimulate the apoptosis of PCa, was investigated in the study. The findings showed a restoration of CES1 expression after treatment with melatonin. In addition, the restoration of CES1 significantly decreased lipid droplet (LD) accumulation, which increased endoplasmic reticulum stress and induced apoptosis, thus repressing CRPC progression. These findings provide unique preclinical information about the role of melatonin in CRPC [131].

Caffeic acid phenethyl ester (CAPE) is a powerful antioxidant that is extracted from the honeybee hive propolis. Research indicates that CAPE treatment inhibits the proliferation of DU145 and PC-3 human prostate cancer cells in a dose-dependent manner [132,133]. The inhibitory effects of CAPE on PC-3 cell development were found to occur within 24 h after CAPE treatment and to accumulate over time. Treatment with 10 M CAPE significantly decreased PC-3 colony development on soft agar. According to flow cytometric analyses, treatment with 3 M to 20 M CAPE decreased the population of S phase cells in PC-3 cells and resulted in G1 cell cycle arrest [132,133]. High doses of CAPE (88–176 M) caused apoptosis in PC-3 cells [134].

Antioxidants such as phytoestrogens have the ability to control inflammation by decreasing the activity of nuclear factor-kappa B (NF-κB). Thus, they serve as AR antagonists, preventing the effects of androgens such as testosterone and DHT [135,136]. Phytoestrogens include isoflavonoids, flavonoids, stilbenoids, and lignans [137]. They have polyphenolic structures and can bind to estrogen receptors in the body to mimic the actions of estrogen. According to a study by Jeong et al. [138], hesperidin, a flavonoid derived from citrus fruits, can suppress the proliferation of androgen-independent PCa cells (PC-3 and DU145) via mitochondrial membrane depolarization, endoplasmic reticulum stress, and reactive oxygen species production. Hesperidin also improves the anticancer effects of the chemotherapy drug cisplatin in PC-3 and DU145 cells. Therefore, antioxidants could be used as therapeutic drugs that specifically target advanced PCa. To the best of our knowledge, no studies have reported on the effects of antioxidants targeting ERα and ERβ in CRPC cases. Therefore, more research is needed to investigate the efficacy of antioxidants in mitigating CRPC by targeting ERs. Table 4 summarizes the application of antioxidants in therapeutic approaches for CRPC.

### 5.5. Advanced Immunotherapy Approach

Several immunoinformatic approaches have been actively improved to inhibit PCa progression and prevent CRPC development. One of these alternatives includes producing vaccines based on the antigenic proteins of PCa. Different immunoinformatic approaches based on the overexpressed antigenic proteins of PCa have been used to create a multi-epitope vaccine. Five antigens specific to PCa were selected for the designing of this vaccine [139]: PSA, prostate-specific membrane antigen (PSMA), prostate stem cell antigen (PSCA), six-transmembrane prostate epithelial antigen (STEAP), and prostatic acid phosphatase (PAP) [140]. PCa is characterized by the upregulation of all of these antigens. Peptide-based vaccines against PCa and cellular metastasis have been developed from these antigens. A study by Park et al. [141] showed that GV1001, a peptide vaccine, reduces Bcl-2 and caspase-3 activity, impairs cell viability, and triggers apoptosis in CRPC cells in vitro. Various stages of angiogenesis, including migration, invasion, and endothelial tube formation, are also suppressed by GV1001. Further, GV1001 reduces the levels of phosphorylated AKT, phosphorylated p65, VEGF, MMP-2, MMP-9, and D31 and increases the expression of TIMP-1 and TIMP-2 in a dose-dependent and dramatic manner. In a CRPC xenograft mice model used in the same study, GV1001 was found to inhibit tumour development and trigger the apoptosis of PCa cells. However, previous findings have indicated that personalized peptide vaccination (PPV) for human leukocyte antigen (HLA)-A24-positive patients with CRPC does not prolong overall survival or PFS. The reduced efficacy of PPV might be due to the target patients having diverse immune cell repertoires, significant tumour burdens, and numerous immune suppressive elements, including increased myeloid-derived suppressor cells (MDSCs) or regulatory T cells, in the tumour microenvironment. Thus, this tumour-associated immunosuppression condition might induce tumour progression and immunotherapy resistance [142].

An alternative technology called proteolysis targeting chimeras (PROTACs) has been developed in current research to promote the inhibition of PCa. PROTACs are also referred to as protein degraders [143] because they irreversibly degrade target proteins via catalytic mechanisms [144]. According to previous research, resistance towards current medication therapies when treating PCa may be overcome by small-molecule PROTACs, which target AR [145]. ARV-110 is a small-molecule PROTAC that degrades AR proteins and has been developed as a potential treatment method for metastatic CRPC [146]. Nevertheless, the application of PROTAC technology in producing ER degraders has only been reported recently for treating breast cancer. In MCF7 and patient-derived xenograft models, PROTACs have been shown to target Erα-inhibited cell growth, induce necrotic cell death, and demonstrate considerable anticancer efficacy by degrading ERα proteins, regardless of whether the ERα-encoding genes were altered [144]. To the best of our knowledge, studies have mostly utilized AR as a biological target for PROTACs, and the exploration of PROTACs that target ER in mitigating CRPC remains limited.

**Figure 3:**
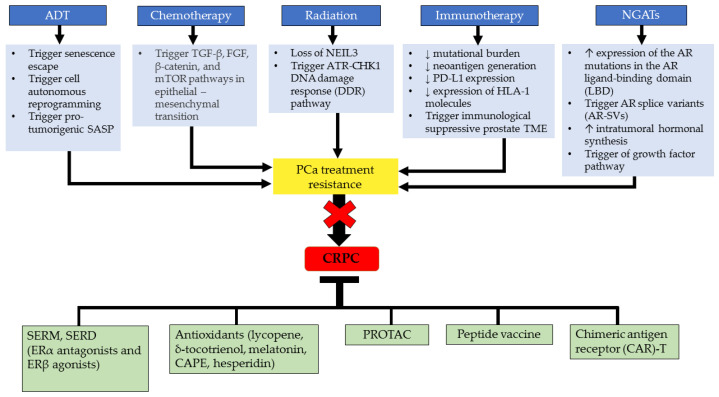
Therapeutic approaches for inhibiting CRPC progression as an alternative in mitigating the treatment resistance that occurs in CRPC. Abbreviations: ADT, androgen-deprivation therapy; CRPC, castration-resistant prostate cancer; AR, androgen receptor; DNA, deoxyribonucleic acid; NGAT, next-generation anti-androgen therapy; PCa, prostate cancer; LBD, ligand binding domain; TGFβ, transforming growth factor beta; FGF, fibroblast growth factor; mTOR, mammalian target of rapamycin; AR-SV, androgen receptor-splice variant; SERM, selective estrogen receptor modulator; SERD, selective ER downregulator; CAPE, caffeic acid phenethyl ester; PROTAC, proteolysis targeting chimeras; CAR-T, chimeric antigen receptor (CAR)-T cell treatment.

Chimeric antigen receptor (CAR)-T cell treatment has had exceptionally effective and long-lasting clinical responses in cancer treatment. CARs are synthetically designed receptors that drive lymphocytes, most often T cells, to identify and destroy cells that express a particular target antigen. However, the scientific discovery of this treatment is rather new, and only a few clinical trials for prostate cancer CAR-T therapy are available to date. A study by Ma et al. [147] demonstrated that CAR-T cells that targeted the PSMA demonstrated a potent PCa-killing impact, and second-generation CAR-T cells outperformed the first generation, presenting a potential method for clinical immune-targeted therapy for CRPC. Figure 3 illustrates the alternative therapeutic approaches for inhibiting the treatment resistance and progression of CRPC.

## 6. Discussion

Hormonal treatment resistance may be caused by the development of alternate intracellular ER signalling, although the exact mechanisms that cause recurrence are yet unclear. The regulation of PCa development into CRPC has been found to be influenced by the estrogen signalling pathway, which is mediated by the estrogen receptors ERα and ERβ. ERα and ERβ are targeted markers of PCa, and researchers’ attention has shifted towards utilizing these markers to inhibit the different stages of the PCa. However, several studies have shown that existing therapeutic applications (e.g., ADT) for PCa have little effect in slowing its progression and its eventual transformation into CRPC. Porter et al. [148] found that a rise in PSA levels after ADT is linked to biochemical recurrence, which leads to CRPC. Therefore, several preclinical and clinical investigations have been conducted to elucidate the roles of ERα and ERβ in CRPC progression. Furthermore, the functionality of ERα and ERβ in therapeutic modulations has been investigated to improve the efficacy of CRPC treatment.

ADT is one of the most common PCa treatments. Even though ADT has been shown to suppress the proliferation of PCa, the development of PCa resistance must also be addressed. The majority of researchers emphasize AR-mediated mechanisms as the underlying cause of CRPC due to ADT. Other mechanisms that can contribute to the development of CRPC include ADT-induced cell senescence, the alternative pathway of steroidogenesis, and the estrogen signalling pathway. Therefore, new strategies need to be used to find appropriate treatment approaches for CRPC patients based on the exact mechanism that leads to the disease.

ERβ may have anticancer activity (onco-suppressor) by altering PCa regulation via ligands [7]. Hence, ERβ has been suggested as a viable therapeutic target for PCa treatment and prevention [149]. A previous study indicated that patients whose tumor cells expressed both nuclear and cytoplasmic ERβ1 had a significantly higher rate of prostate cancer-specific death [150]. Di Zazzo et al. [7] found that only ERβ1 is functional among the ERβ isoforms, while the other ERβ isoforms regulate its activity. Therefore, ERβ activity may be influenced by ERβ1 expression and the ERβ isoform ratio. Nevertheless, several ERβ isoforms were found to exhibit opposite functionality in PCa [47,151]. In higher grade PCa, including CRPC, ERβ1 was found to be lost, whereas ERβ2 is the main expressed ERβ isoform in the advanced stage of PCa [152]. Differentially expressed ER isoforms have been found to promote PCa progression, implying that the duality of ER actions must be considered when developing better treatment initiatives for CRPC.

The actions of both ERα and ERβ in PCa development are associated with estrogen-related receptors (ERRs). ERRs and ERs are positively coexpressed in normal prostate and PCa cells. The three subtypes of ERRs are ERRα, ERRβ, and ERRγ [153]. The DNA-binding domains and LBDs of the ERRs are highly homologous to those of ERs, but ERRs do not bind to estrogen [154]. Nevertheless, there is crosstalk and overlap between ER and ERR mechanisms since they bind to the same response elements, such as EREs [155]. Therefore, it has been hypothesized that ERRs transactivate groups of genes that are likewise targeted by ERs due to their similar properties of binding to EREs and ERE-related response elements [8,156]. Moreover, several in vitro studies have revealed that ERα and ERRα can form a heterodimer through direct protein–protein interactions [157,158]. In fact, ERRα also possesses the ability to promote cell proliferation in CPRC, similar to ERα. Rapid bone tumour growth in CRPC cases is caused by elevated ERRα levels in tumour cells [159]. A study by Zhou et al. [160] demonstrated that castration-relapse VCaP–CRPC xenograft models and metastatic CRPC tissues both exhibit an upregulated expression pattern for ERRα, which may contribute to the progression of CRPC. On the other hand, it has been illustrated that ERβ expression is related to ERRβ expression and that ERRβ levels are inversely correlated with the S-phase fraction in the cell cycle. This suggests that the orphan receptor slows down cellular proliferation or promotes cellular differentiation. However, this significant association between ERβ and ERRβ has only been seen in cases of breast cancer [154]. Nevertheless, both androgen-dependent and androgen-independent PCa cells have been found to exhibit suppressed cell proliferation when ERRβ or ERRγ is overexpressed, indicating that these receptors have antiproliferative or tumour-suppressive effects in both local and advanced PCa [161]. This implies that ERRβ and ERRγ exhibit the same antioncogenic properties as ERβ in CRPC. More studies on ERs as well as ERRs need to be carried out to obtain clear insights and develop a therapeutic approach that modulates the interaction between these two receptors in CRPC.

ERβ exhibits anticancer activity (onco-suppressor) by altering PCa regulation via ligands [7]. Therefore, ERβ has been suggested as a viable therapeutic target for PCa treatment and prevention [149]. A previous study has indicated that patients whose tumour cells express both nuclear and cytoplasmic ERβ1 have a significantly higher rate of prostate cancer-specific death [150]. Di Zazzo et al. [7] found that only ERβ1 is functional among the ERβ isoforms, whereas the other ERβ isoforms regulate its activity. Therefore, ERβ activity may be influenced by ERβ1 expression and the ERβ isoform ratio. Nevertheless, several ERβ isoforms have been found to exhibit the opposite functionality in PCa [47,151]. In higher-grade PCa, including CRPC, a loss of ERβ1 has been observed, whereas ERβ2 has been reported to be the main expressed ERβ isoform in advanced PCa [152]. Differentially expressed ER isoforms have been found to promote PCa progression, implying that the duality of ER actions must be considered to develop better treatment initiatives for CRPC.

Numerous medications are being developed for the treatment of CRPC, with the aim of delaying the disease’s progression and increasing patient survival. Apart from targeting the AR, causing cell cycle arrests, and enhancing patients’ immune system, the available drugs also target ER signalling, specifically ERα and ERβ. The adverse effects of estrogen are exhibited when this hormone binds to ERα, which plays an oncogenic function in PCa development, inflammation, and cell proliferation. Even though studies have reported on therapies that target the proliferative effects of ERα in PCa via ERα antagonists [11,110,162], ERα-targeted treatments that inhibit CRPC remain limited. Only one SERM drug, toremifene, has exhibited antagonistic action against ERα in an androgen-independent cell line [110,163]. Toremifene is a nanotechnology drug that competes with E2 in binding to ERα, leading to a reduction in the tumourigenicity of PCa [110]. ERα is primarily localized at the nuclei of cardiac myocytes and is the dominant estrogen receptor in these cells. ERα regulates transcription in cardiac myocytes; therefore, inhibition of this receptor might affect the functionality of cardiac myocytes [162]. Our literature search revealed a lack of studies on the effects of drugs that target ERα in CRPC. The distribution and functionality of ERα in other organs may also be affected during the treatment of CRPC. However, this estrogen receptor can be targeted during the treatment of CRPC using SERM and nanotechnology approaches, although more research is needed to develop effective ERα antagonists.

In contrast to ERα, ERβ exhibits antiproliferative activity, delaying the progression of PCa. The use of ERβ agonists such as 8-VE2 and DPN has been proven to increase the antiproliferative actions of ERβ in PCa [11]. Short-term ADT (AA) acts as an AR inhibitor and has been found to enhance ERβ expression in advanced PCa [163]. There is a window of opportunity during which ADT, when used in a specific way and at the right time, can help ERβ expression in advanced PCa. Therefore, the treatment period when using an AR inhibitor as an alternative ERβ agonist must be taken into account, as it plays a vital role in conducting successful therapy. To the best of our knowledge, the effectiveness of this synergistic therapeutic approach (combination of ERβ agonists and ADT) in CRPC is limited. Therefore, more research needs to be carried out to evaluate the efficacy of combining these therapeutic agents. Moreover, the target selection of ERβ agonists must be evaluated carefully to prevent the stimulation of several ERβ isomers (ERβ2, ERβ3, ERβ4, and ERβ5) from activating tumour proliferation in PCa cases.

Commercial drugs are typically used in combination, and this has been shown to reduce PSA levels by more than 50% while also increasing patient survival in most CRPC cases. The efficacy of antioxidants in suppressing tumour progression in CRPC has also been reported. Antioxidants suppress CRPC progression by inducing cell cycle arrest, stimulating the endoplasmic reticulum stress pathway, and stimulating apoptosis via CES1 [131]. Phytoestrogen and E2 also have similar effects in inhibiting the progression of CRPC [138]. However, the inhibitors of certain pathways involved in CRPC progression and its downstream signalling partners have exhibited adverse effects after treatment [114]. Thorough modulations of these drugs are crucial to reducing the underlying side effects. To the best of our knowledge, most commercial drugs and natural products, such as antioxidants, have effects against CRPC; however, due to a lack of evidence regarding their effects on ERα and ERβ, it is unclear whether these drugs are fully dependent on ER pathways.

Vaccination approaches using peptide vaccines produced from antigens of PCa proteins are currently being clinically tested, and most studies so far have been carried out at the trial stage. Noguchi et al. [142] obtained nonsignificant findings on the overall survival of CRPC patients treated with peptide vaccines, suggesting that thorough initial health screening is crucial to determine the tumour-associated immunosuppression statuses of patients prior to vaccination and thereby ensure successful treatment. PROTAC technology, which focuses on the utilization of AR, should be further explored by targeting ER, as it has the potential to mitigate PCa progression by modulating alternative estrogen pathways.

The utilization of treatments involving high doses of SERMs and the use of ERβ agonists that lack selectivity for each of the ERβ isomers exhibit off-target effects, resulting in several discrepancies between the expression of ERs and the effectiveness of SERMs for CRPC treatment [42,156,157]. Furthermore, there is an ongoing debate regarding how precisely the CRPC models (such as genetically modified mice, xenografts, and cell culture models) that are currently being employed mimic the crucial aspects of the human prostate. Lafront et al. [37] demonstrated that traditional CRPC models are ineffective for studying the estrogen signalling pathway and suggested that this could contribute to the variations seen in ER expression in cultured cells. Therefore, there is a crucial need to find suitable CRPC models that express biologically relevant levels of ERα and ERβ to accurately evaluate the functions of the estrogen signalling pathway in PCa progression to CRPC.

## 7. Conclusions

We have summarised that ERα exhibits proliferative action, whereas ERβ plays an anti-proliferative role in CRPC. The majority of the researchers suggested that the castration resistance mechanisms are mainly AR-dependent pathways. However, other mechanisms, such as ADT-induced senescence, the alternative pathway of steroidogenesis, and the ER signaling pathway, are also involved in CRPC development. A deeper knowledge of the resistance mechanisms behind successive transitions from PCa to more advanced stages after ADT is necessary for the development of effective treatments for CRPC. Furthermore, our findings lay the groundwork for further research into the involvement of ERα and ERβ in PCa that results in its castration stage, CRPC, and emphasises the significance of ER distribution in various study models. Co-targeting these receptors for CRPC prevention via the modification of SERM and antioxidants is crucial to improving future therapeutic approaches.

## Figures and Tables

**Table 1:** Expression of ERα and ERβ in castration-resistant prostate cancer study model.

Study Model	Estrogen Receptor	Findings	Author
Human PC-3 cell line	ERβ	ERβ regulates the cell cycle of PCa by controlling the expression of CCND1 (*p* < 0.05)	[63]
Tissue microarray consisting of PCa samples from CRPC patients (*n* = 101)	ERα ERβ	ERα ↓ ERβ1 ↑ (*p* < 0.05)	[57]
Human PC-3 cell line	ERα	ERα ↑	[37]
Human PC-3 and LNCaP cell lines	ERβ1	ERβ1 and 3β-Adiol repress mesenchymal characteristics VEGF-A and EMT expression ↓ (*p* < 0.05) TGF-β and hypoxia reduced expression of ERβ1	[55]
Blood-derived RNA samples from CRPC patients (*n* = 42)	ERα ERβ	Detection of four mutations of ERα (E380Q, L536Q, Y537S and D538G) ERβ splice variant concentrations ↓	[45]
Public genomic datasets from patients with metastatic or advanced prostate cancer (*n*= 150) and patients with early prostate cancer (*n* = 492)	ERα ERβ	The prevalence of ERα and ERβ mutations: 3% in patients with metastatic or advanced PCa; 2% with early stage of PCa	[85]
Primary tumor tissue from radical prostatectomy (RP) patients (*n* = 535)	ERβ	ERβ was correlated with decreased time to biochemical failure (BF) (*p* = 0.002)	[46]
Human PC-3 (derived from bone metastasis) and DU-145 (derived from brain metastasis) cell lines	ERα ERβ	ERα activation via PPT (ERα-selective agonist) ERβ activation via DPN (ERβ-selective agonist) Both ERα and ERβ increase the migration and invasion of PC-3 cell lines	[35]

Abbreviations: ↑: increase; ↓: decrease; PPT: propylpyrazoletriol; DPN: diarylprepionitrile; CCND1: cyclin D1; EMT: epithelial-mesenchymal transition; VEGF-A: vascular endothelial growth factor A; biochemical failure: BF.

**Table 2:** Application of SERMs in castration-resistant prostate cancer treatments.

Treatment	Number (n)	Findings	Author
ADT+ toremifene 300–640 mg/m^2^	15	No cancer inhibitory effect	[109]
Raloxifene 60 mg	13	Partial effect (5 of 13 patients)	[108]
Fulvestrant 500 mg, 250 mg	20	No patients reduced PSA by >50%	[111]
Raloxifene (60 mg) + bicaltamide (50 mg)	18	Partial effect (4 of 18 patients)	[107]
Concentrations of 5 µM and 25 µM of ERβ-selective agonist, 8-VE2, and 8β-VE2 in VCaP cells	n/a	The expression of AR protein ↓ by 52% AR mRNA ↓ by 40%	[112]
Nano-targeted delivery of toremifene (1.0 to 100 μM) in PC3M cancer cell line	n/a	Prostate tumor growth ↓ by blocking ERα	[110]

Abbreviations: ↑: increase; ↓: decrease; PSA: prostate-specific antigen; ADT: androgen deprivation receptor, AR: androgen receptor; mRNA: messenger ribonucleic acid.

**Table 3:** Application of selective inhibitors in castration-resistant prostate cancer treatments.

Treatment	Number (n)	Findings	Author
Tamoxifen (20 mg/kg) + ISA-2011B (20 mg/kg) in PC-3 cells	25	ERα expression significantly ↓ (*p* < 0.001)	[124]
1 M of PI3K specific inhibitors (Wortmannin), 200 nm of AKT inhibitor (MK2206) and 5 nM of SRC-family kinase inhibitor (PP2)	n/a	Size and number of colony of PC-3 cell line significantly ↓ (*p* < 0.05)	[125]
mTOR inhibitor (MLN0128)	9	8 of 9 patients quit taking the medication before the endpoint and experienced Grade 3 adverse effects, such as mucositis, rash, pain, delirium, and dyspnea	[122]
Pan-PI3K inhibitor (BKM120) +/− enzalutamide		The median progression-free survival was 1.9 months (in combination with enzalutamide) as opposed to 3.5 months	[117]
Akt inhibitor (AZD5363) + enzalutamide in mCRPC	13	3 of 13 patients (23%) experienced a PSA decrease of more than 50% Patients experienced side effects: rash (20%) and hyperglycemia (26.7%)	[121]

Abbreviations: ↑: increase; ↓: decrease; PSA: prostate-specific antigen.

**Table 4:** Application of antioxidants in castration-resistant prostate cancer treatments.

Treatment	Findings	Author
Lycopene	Growth inhibition on DU145 cells ↑ Docetaxel + lycopene: tumor regression ↑, with a 38% increase in antitumor efficacy (*p* < 0.05)	[129]
δ-Tocotrienol	δ-TT exerts a cytotoxic/proapoptotic activity in CRPC cells	[130]
Melatonin	Inhibition of tumor growth CES1 expression ↑	[131]
CAPE	Proliferation of DU-145 and PC-3 cells dose-dependently ↓	[132]
CAPE	Proliferation of DU-145 and PC-3 cells dose-dependently ↓	[133]
CAPE	Induced apoptosis in PC-3	[134]
Hesperidin	Suppressed the proliferation of androgen-independent PC-3 and DU145 Improved the anticancer effects of the chemotherapy drug cisplatin in PC-3 and DU145 cells	[138]

Abbreviations: ↑: increase; ↓: decrease; CAPE: caffeic acid phenethyl ester; CES1: carboxylesterase 1; δ-TT: δ-tocotrienol.

## Data Availability

Not applicable.

## Abbreviations

3D	Three-dimensional
3β-Adiol	5α-Androstane-3β,17β-Diol
AA	Abiraterone acetate
ADT	Androgen deprivation therapy
AE	Adverse events
AF1	Activation function 1
AF2	Activation function 2
AR	Androgen receptor
AR-SV	Androgen receptor-splice variant
ATP	Adenosine triphosphate
BAD	Bcl-2-associated death
BF	Biochemical failure
BMS	Bone marrow stromal
CAPE	Caffeic acid phenethyl ester
CAR-T cell	Chimeric antigen receptor- t cell
CCND1	Cyclin D1
CES1	Carboxylesterase 1
CHD1	Chromodomain-helicase DNA-binding protein
CRPC	Castration-resistant prostate cancer
CYP17A1	Cytochrome P450 17A1
CYP191A1	Cytochrome P450 191A1
DBD	DNA-binding domain
DDR	DNA damage response
DHEA	Dehydroepiandrosterone
DHEA-S	Dehydroepiandrosterone sulfate
DHT	Dihydrotestosterone
DNA	Deoxyribonucleic acid
DPN	Diarylprepionitrile
DSBs	Double-strand breaks
E2	Estradiol
EMT	Epithelial-mesenchymal transition
ENZ	Enzalutamide
ER	Estrogen receptor
EREs	Estrogen response elements
ERR	Estrogen-related receptor
ERα	Estrogen receptor alpha
ERβ	Estrogen receptor beta
ESR1	Estrogen receptor 1
ESR2	Estrogen receptor 2
FGF	Fibroblast growth factor
FOX	Forkhead box protein
FOXO	Forehead box O
GEM	Genetically engineered mouse
GnRH	Gonadotropin-releasing hormone
HIF-α	Hypoxia-inducible factor 1-alpha
HLA	Human leukocyte antigen
ICIs	Immune checkpoint inhibitors
KO	Knocked-out
LBD	Ligand-binding domain
LD	Lipid droplet
LH	Luteinizing hormone
LHRH	Luteinizing hormone-releasing hormone
mCRPC	Metastasis castration-resistant prostate cancer
MDSC	Myeloid-derived suppressor cells
mHSPC	Metastatic hormone-sensitive prostate cancer
MMP12	Matrix metalloproteinase 12
mRNA	Messenger ribonucleic acid
mTOR	Mammalian target of rapamycin
NF-κB	Nuclear factor-kappa beta
NGATs	Next-generation anti-androgen therapies
OS	Overall survival
PAP	Prostatic acid phosphatase
PCa	Prostate cancer
PCSLC	Prostate cancer stem-like cell
PDC	Poorly differentiated carcinoma
PDE	Patient-derived explant
PDKI	Phosphorylation of phosphoinositide-dependent kinase
PDO	Patient-derived organoids
PDX	Patient-derived xenografts
PDX	Patient-derived xenograft
PDXOs	Patient-derived xenograft-derived organoids
PFS	Progression-free survival
p-mTOR	Phosphorylated mTOR
PPT	Propylpyrazoletriol
PPV	Personalized peptide vaccination
PROTAC	Proteolysis targeting chimeras
PSA	Prostate-specific antigen
PSCA	Prostate stem cell antigen
PSMA	Prostate-specific membrane antigen
PTEN	Phosphatase and tensin homolog
RECIST	Response evaluation criteria in solid tumors
RNA	Ribonucleic acid
RP	Radical prostatectomy
SASP	Senescence-associated secretory phenotype
SERDs	Selective estrogen receptor down regulators
SERMs	Selective estrogen receptor modulators
SPOP	Speckle-type poz protein
SRC	Steroid receptor coactivator
SRC-PI3K-AKT-mTOR	Steroid receptor coactivator/phosphoinositide 3-kinase/akt/mammalian target of rapamycin
SSBs	Single-strand breaks
STEAP	Six-transmembrane prostate epithelial antigen
TCF/LEF	T cell factor/lymphoid enhancer factor transcription factors
TGF-β	Transforming growth factor-beta
TME	The tumour microenvironment
TRAMP	Transgenic adenocarcinoma of the mouse prostate
VCaP	Vertebral cancer of the human prostate
VEGF-A	Vascular endothelial growth factor A
WLS	Wntless
δ-TT	Delta-tocotrienol
